# Primary versus secondary intraocular lens implantation following removal of congenital/developmental cataracts: outcomes after at least 4 years

**DOI:** 10.55730/1300-0144.5560

**Published:** 2023-02-11

**Authors:** Ayşin Tuba KAPLAN, Sibel ÖSKAN YALÇIN, Ayşe Yeşim ORAL AYDIN

**Affiliations:** 1 Department of Ophthalmology, Kartal Dr. Lütfi Kırdar City Hospital, University of Health Sciences, İstanbul, Turkey; 2 Department of Ophthalmology, Faculty of Medicine, Afyonkarahisar Health Sciences University, Afyonkarahisar, Turkey

**Keywords:** Binocular vision, IOL implantation, myopic shift, strabismus, visual acuity

## Abstract

**Background/aim:**

The aim of this study is to evaluate the long-term outcomes of primary and secondary intraocular lens (IOL) implantation following removal of congenital/developmental cataracts.

**Materials and methods:**

One hundred and forty-four patients aged under 16 years who were followed up between 2003 and 2021 were analyzed retrospectively. The long-term results of children who underwent surgery before 2 years of age for congenital or developmental cataracts and underwent secondary IOL implantation after 2 years of age and those who underwent cataract surgery with primary IOL implantation after 2 years of age were compared. Patients with traumatic, secondary cataracts and cataracts due to ocular anomalies were not included in the study.

**Results:**

We evaluated 64 patients (mean age 9.5 ± 4.5 years) with secondary IOL implantation and 80 patients (mean age 12.8 ± 4.1 years) with primary IOL implantation in the study. Distance and near best-corrected visual acuities were significantly better in the primary IOL group than the secondary IOL group (p < 0.001). Incidence of strabismus after primary IOL surgery was significantly lower and presence of binocular vision was more often than the secondary IOL group (p = 0.002). There was no significant difference between the two groups in terms of refraction and myopic shift (p = 0.242, p = 0.172, respectively). Mean refractive changes were significant in unilateral cases of secondary IOL group and primary IOL group (p = 0.013, p = 0.049, respectively) and myopic shift was also greater in both groups of unilateral cases than the fellow eyes (p = 0.023, p = 0.012, respectively).

**Conclusion:**

Visual outcomes and binocular vision were better, and the incidence of strabismus was also much less in the primary IOL group.

## 1. Introduction

Congenital/developmental cataract can cause blindness or vision loss in childhood. It is responsible for approximately from 5% to 20% of all vision loss in children worldwide [1]. The long-term results of pediatric cataract surgery depend on many factors, such as the age of onset, the age of surgery, associated ocular anomalies and complications [2,3]. Various factors determine the probability of functional and morphological success after surgery. According to Wu et al., treatment of cataract in children is one of the most difficult and costly interventions in ophthalmology [4].

Childhood cataracts have unique challenges compared to adult cataracts. These difficulties can be described by the fact that the surgical technique needs more care, complication types and management are more specific to that age group and the predictability of refractive error is more variable than adults [5]. The most common problems after the surgery are aphakic glaucoma, anisometropic amblyopia, strabismus, loss of binocular vision, and posterior capsule opacification [6]. The earliest recommended age for IOL implantation in children is still controversial since the children’s eyeball that has undergone cataract surgery continues to develop and shows refractive changes in childhood and early adulthood. Intraocular lens (IOL) implantation is accepted as the gold standard by most surgeons after the age of 2 in children who have had cataract surgery while IOL implantation is standard for adult patients [5,7].

The aim of this study is to evaluate the long-term results of cataract surgery in pediatric patients with primary and secondary IOL implantation.

## 2. Materials and methods

This retrospective study was conducted at ophthalmology department from January 2003 to December 2021. Patients who were operated before 2 years of age for congenital or developmental cataract and underwent secondary intraocular lens implantation after 2 years of age, and patients older than 2 years of age with developmental cataracts who had primary lens implantation were included in the study. Patients with secondary or traumatic cataracts and cataracts due to ocular anomalies were excluded in the study. The minimum follow-up period was 4 years in both groups. The study was confirmed by the ethics committee of Kartal Dr. Lutfi Kırdar City Hospital and was conducted in accordance with the Helsinki Declaration. Informed consent was obtained from the families of all patients. All information about patients such as age at admission, type of cataract, laterality, associated ocular anomalies, visual acuity, follow-up time, complications, and additional surgeries were collected from medical and surgical records. Cataracts diagnosed in children younger than 6 months were defined as congenital cataracts and cataracts diagnosed after 6 months of age were defined as developmental cataracts.

Visual acuity (VA) was evaluated according to the age of the child. In children under 3 years of age the central, steady and maintained fixation (CSM) method was used, and the Lea symbols, E chart or Snellen’s acuity chart was used in children aged 3–6 years. Ophthalmic examination was done by biomicroscope or operating microscope and fundus imaging was done by indirect ophthalmoscope or B scan ultrasonography in the presence of dense media opacities. IOL power was calculated with the SRK/T formula [8]. Axial length (Sonomed, E-ZSCAN AB 5500+) and keratometry (Nidek, Aichi, Japan) were measured manually in young children and Lenstar 900 biometer (Alcon; Haag-Streit, Switzerland) could only be used in well cooperative children for the IOL power calculation. In order to prevent postoperative myopic shift, postoperative hyperopic refraction was targeted according to the age of the child (+4 D for under 2 years old, +3 D for a 2 to 3 years-old, +2 D for a 4 to 5 years old, +1 D for a 6-year-old, and emmetropia for older ages).

All surgeries were performed by one of the surgeons (AYO, ATK) under general anesthesia. The surgical technique included; anterior capsulorhexis after 2.8 mm corneal and 2.0 mm side port incisions, lens aspiration or phacoemulsification (if needed), posterior capsulorhexis and anterior vitrectomy in all children under 6 years of age. If there was no mental retardation or cooperation disorder in children older than 6 years, posterior capsulorhexis was not performed and YAG capsulotomy performed after the surgery when necessary. IOL implantation was not performed in any children under the age of 2, and visual rehabilitation was performed with glasses or contact lenses after the surgery. Secondary IOL implantation was preferred in all children after 2 years of age, and primary IOL implantation was performed in the same session in children diagnosed as having developmental cataract after 2 years of age. It was aimed to implant hydrophobic acrylic IOL in the bag, but if it was not safe, 3-piece acrylic IOL was placed in the ciliary sulcus. Sulcus implantation was performed in cases where the posterior capsulorhexis was large and the IOL was not safe in the bag or in capsular phimosis cases that could not be differentiated. Complications during and after surgery were recorded. Postoperatively, topical corticosteroids, antibiotics, cycloplegic, and mydriatic agents were used as treatment for all patients. At each follow-up, patients were evaluated for distance and near best-corrected visual acuity (BCVA), refractive status (spherical equivalent, SE), binocular vision (Titmus Stereo test), nystagmus, strabismus, and amblyopia.

### 2.1. Statistical analysis

Statistical analysis was performed using SPSS Statistics program v. 26 (IBM Corporation, Armonk, New York, USA). The data were given as mean ± standard deviation, median, and interquartile range. The normal distribution of the data of numerical variables was evaluated with the Shapiro Wilk test of normality. Homogeneity of variances was evaluated with Levene’s test. Comparisons were made using the t-test and Mann-Whitney U test. LogMAR and SE values of the groups were compared using linear mixed (fixed effect + random effect) models. Bonferroni correction was used to compare the main effects in linear mixed models. Chi-square and Fisher’s exact tests were used to compare categorical variables between groups. A p value less than 0.05 was accepted statistically significant.

## 3. Results

We evaluated 242 eyes of 144 patients in the study. Sixty-four patients (31 females and 33 males, mean age 9.5 ± 4.5 years, 111 eyes) with congenital/developmental cataracts who underwent surgery before 2 years of age, followed by secondary IOL implantation, and 80 patients (25 females and 55 males, mean age 12.8 ± 4.1 years, 131 eyes) aged over 2 years with developmental cataracts who underwent primary IOL implantation (Table 1).

In the secondary IOL group, the mean age at the time of cataract surgery was 0.4 ± 0.3 years and the mean age at the time of IOL implantation was 2.8 ± 0.4 years. The median follow-up was 8.0 (range, 4–19) years. Twenty-nine (45.3%) patients had strabismus (esotropia in 14, exotropia in 14 and hyperopia in one patient) and 29 (45.3%) patients had nystagmus before the surgery. Thirty three patients underwent early surgery (age ≤8 weeks) and 13 (38%) had nystagmus at the most recent follow-up, 31 patients underwent late surgery (age >8 weeks) and 16 (51%) had manifest nystagmus at the most recent follow-up. There was no significant difference between the timing of cataract surgery and the development of postoperative nystagmus (p = 0.44). Postoperative VA was 20/40 or less in 8 of 35 patients without nystagmus and 16 of 29 patients with nystagmus. Therefore, postoperative VA ≤20/40 was significantly more common in patients with nystagmus than in patients without nystagmus (p < 0.05). Postoperative visual acuity of 20/40 or less was not significantly different in patients with strabismus compared to those without in both groups (p > 0.05) (Table 2). Preoperative or postoperative presence of strabismus was associated with a high probability of accompanying nystagmus in secondary IOL group, there was no high correlation between presence of strabismus and nystagmus in the primary IOL group (Table 3).

No significant complications were observed during surgery. Capsular bag implantation was performed in 65.7% of patients. Postoperative complications were present in 30 (27.0%) eyes. The most common ocular complications were inflammatory membrane (9.0%) and glaucoma (7.2%). Secondary additional surgery was required in 28 eyes (25.2%). Two eyes underwent trabeculectomy and the other 6 eyes with glaucoma were treated using antiglaucomatous therapy. Strabismus surgery was performed in 14 (12.6%) patients. The complications and surgeries in both groups are shown in Table 4. At the last examination, the distance and near BCVA values were 0.612 ± 0.051 LogMAR and 0.450 ± 0.031 LogMAR, respectively, and the last follow-up refraction (SE) was −0.834 ± 0.410 D (Table 5). Sixty-four (57.7%) eyes had myopia, and 47 (42.3%) eyes were hyperopic (Table 6). Thirty-three (51.6%) patients had strabismus at the last visit postoperatively. Esotropia was present in 16 (48.4%), exotropia in 14 (42.2%), and hypertropia in 3 (9.1%) patients. Stereopsis was present in only three (4.7%) patients and occlusion therapy was performed in 38 (59.4%) patients.

In the developmental cataract surgery group, 49 patients underwent primary IOL implantation with posterior capsulorhexis and anterior vitrectomy under the age of 6 years. The mean age at the time of cataract surgery was 5.9 ± 3.3 years and the median follow-up was 5.0 (range, 4–16) years. Before surgery, four patients (5.0%) had strabismus (3 esotropia, 1 exotropia) and three (3.7%) had nystagmus. Postoperative VA was 20/40 or less in 12 of 77 patients without nystagmus and 3 of 3 patients with nystagmus. Therefore, postoperative VA ≤ 20/40 was significantly more common in patients with nystagmus than in patients without nystagmus (p < 0.05) (Table 2).

No complications were observed in any patients during surgery. Capsular bag implantation was performed in 92.1% of patients. Sulcus implantation was performed in cases (7.9%) where the IOL was unstable due to the large posterior capsulotomy. Postoperative complications were present in 24 (18.3%) eyes. The most common postoperative ocular complications were visual axis opacity (VAO) (9.1%) and inflammatory membrane (5.3%). Secondary additional surgery was required in 17 (12.9%) eyes. Visual axis opacity was removed by Nd:YAG laser in eight (6.1%) eyes and membranectomy was performed in six (4.5%) eyes: none required glaucoma surgery (Table 4). At the last examination, the distance and near BCVA values were 0.268 ± 0.041 LogMAR and 0.305 ± 2.1 LogMAR, respectively. The mean refraction was −0.148 ± 0.415 D in the last follow-up. Myopic shift was present in 64 (48.95%) eyes, and 67 eyes were hyperopic. Strabismus was present in 20 (25%) patients (esotropia in 7 (35%), exotropia in 12 (65%) and hyperopia in 1 (5%) patient), five of whom had strabismus surgery. Stereopsis was present in only 19 (23.7%) patients. Occlusion therapy for amblyopia was performed in 60 (75%) patients.

Distance and near BCVA were significantly better in the primary IOL group than in the secondary IOL group (p < 0.001). The incidence of strabismus after primary IOL surgery was significantly lower and the presence of stereopsis was more frequent than in the secondary IOL group (p = 0.002). There was no significant difference between the two groups in terms of refraction and myopic shift at the last follow-up (p = 0.242, p = 0.172 respectively).

In the unilateral surgical cases, mean refraction and myopic shift were statistically different compared with the healthy other eyes in both groups. Mean refractive changes were significant in unilateral cases (p = 0.013 and p = 0.049, respectively), and myopic shift was greater in both unilateral cases than in the healthy other eyes (p = 0.023 and p = 0.012, respectively) (Table 7).

## 4. Discussion

Implantation of an IOL provides a continuous correction of refraction after cataract surgery; however, the timing of the implantation is controversial. Extreme myopic refractive errors can develop in adulthood because axial elongation and consequent large myopic shift occur after primary IOL implantation in the early stages of life. Therefore, many surgeons prefer to leave children’s eyes with residual hyperopia, predicting myopic shift, but as the results from the Infant Aphakia Treatment Study (IATS) demonstrate, this refractive change is variable and at times unpredictable [9]. Because the socioeconomic status of the families is quite low in the environment we live in, the vast majority cannot maintain the consistent use of contact lenses and glasses. Therefore, secondary IOL implantation was performed at the earliest period after the age of 2 years. Most studies supported later secondary IOL implantation in children [10–13]. In the IATS, secondary IOL surgery was performed after 5 years of age, except for patients with aphakic lenses with inconsistent use of contact lenses and glasses [9].

In our study, the rate of capsular bag implantation was 65.7% in the secondary IOLs and 92.1% in the primary IOLs. Secondary and primary IOL surgeries with capsular bag implantation have been described with low complication rates and high visual outcomes. Capsular bag implantation is performed whenever possible because implantation of the ciliary sulcus carries a higher risk of prolonged iris contact and inflammation, pigment dispersion, glaucoma, and IOL decentration [12,14,15].

In the present study, the final distant and near BCVA were significantly better in the primary IOL group than in the secondary IOL group (p < 0.001). Studies have shown better visual outcomes in eyes with primary IOL implantation because IOLs have the advantage of providing a stable retinal image with full-time correction and minimal anisocoria [16]. The age at cataract extraction and compliance with postoperative treatment are expected to be major factors in the outcome of visual rehabilitation in the secondary IOL group. Especially, in the aphakia period, irregular use of glasses or contact lenses and noncompliance with the occlusion treatment cause VA not to reach the desired level. In the IATS, the authors found no associations or changes in final VA among eyes with secondary IOL implantation compared with eyes that remained aphakic [9]. However, the families in their study received financial and emotional support for patches and contact lenses. Myopic shift has been reported frequently after pediatric cataract surgery [17–20]. In the present study, we found myopic shift in both groups, and the difference was not significant (p = 0.172). In their longitudinal study, Crouch et al. reported myopic shift that persisted into early adolescence after the cataract surgery [6]. Although previous reports attributed myopic shift in operated eyes to excessive axial elongation resulting from amblyopia and visual deprivation, Enyedi et al. stated that they did not believe that the myopia tendency in pseudophakia was the result of excessive elongation, but instead to be the result of normal eye growth with a constant IOL power [18,21,22]. Moreover, Dahan and Drusedau reported that pseudophakic eyes showed the greatest axial elongation in the first 2 years and continued to grow more slowly until 8 years of age, as in normal phakic eyes [19]. In the IATS, axial length growth in operated eyes was similar to that of nonoperated eyes and there was no significant difference between aphakic eyes and the pseudophakic eyes in 5 years of follow-up. They reported greater axial elongation in eyes with glaucoma and secondary opacification and emphasized that the most important factors affecting axial elongation in pediatric patients were early glaucoma development and secondary VAOs requiring surgery [23,24].

In our study, unilateral cases were evaluated and compared with nonoperated eyes in terms of refraction errors and myopic changes. There were significant differences in both groups in the refraction error and myopic shift between the operated and nonoperated eyes (p = 0.013, p = 0.049, p = 0.023, and p = 0.012, respectively). This was explained in the literature by the fact that the IOL power was stable in pseudophakic eyes, whereas the normal phakic lens preserved accommodation and compensated for developmental prolongation [6,18]. McClathey et al. argued that myopic shift in an operated eye was an optical phenomenon; as the eye grows, the anterior movement of the IOL away from the retina causes myopic shift by itself [25]. We cannot discuss how axial elongation affected refractive status in operated eyes because we did not measure the axial length of eyes in both groups.

In long-term follow-up, the presence of strabismus both before and after surgery was greater in the secondary IOL group than the primary IOL group (45.3%, 5.0% preoperatively and 51.6%, 25.0% postoperatively, respectively), and the need for strabismus surgery was greater in the secondary IOL group. Tartarella et al. reported the rate of preoperative strabismus as 55.55% in congenital cataracts and 47.77% in developmental cataracts, and they concluded that earlier onset of visual deprivation led to greater involvement of visual pathways [26]. In an ocular alignment study, strabismus was reported in 27 (66%) of 41 children postoperatively and it was shown that congenital cataracts were correlated with a greater risk of developing strabismus than developmental cataracts [27]. In addition, it was emphasized that there was a significant risk for the development of strabismus in patients in whom visual deprivation remained longer than 6 weeks. In our study, postoperative visual acuity of 20/40 or less was not significantly different in patients with postoperative strabismus compared to those without in both groups. Magli et al. speculated that strabismus in pediatric patients with cataracts could be associated with many factors that could affect visual perception: decreased vision in one eye due to amblyopia, unequal visual acuity in bilateral cataracts, loss of binocular vision, and anisometropia [28].

We could not find significant difference between the timing of cataract surgery and the development of postoperative nystagmus in the secondary IOL group. Young et al. compared early versus late surgery and they found no statistically significant difference between the groups and the development of postoperative nystagmus. Similarly, Robb and Petersen reported 51 patients who underwent early surgery and of the 51 patients 19 (37%) developed nystagmus; therefore, they concluded that early surgery did not seem to prevent its development [29,30]. During the follow-up period, nystagmus became latent in one patient in the primary IOL group and improved in four patients during the aphakic period in the secondary IOL group, most likely due to improved visual function. In many studies, age at surgery time and duration of vision loss have also been suggested as risk factors for the development of nystagmus [31–34]. In our study, we found a strong negative correlation between the presence of preoperative nystagmus and VA, but Bradford and Young did not find correlation between the presence of preoperative nystagmus and VA in their study. They concluded that the presence of preoperative nystagmus was not a poor prognostic indicator [29,35].

The most common ocular complications were secondary membrane (9.0%) and glaucoma (7.2%) in the secondary IOL group, and PCO (9.1%) and secondary membrane (5.3%) in the primary IOL group. Secondary membrane occurred in eight (7.2%) eyes after the first surgery in the secondary IOL group. Knight-Nanan et al. reported anterior chamber reaction in seven eyes (29.0%) aged under 22 months with primary IOL implantation and Ram et al. reported it in only four eyes (9%) aged under 2 years with primary IOL implantation. Similarly, Wood et al. reported secondary membrane formation in 7% of their secondary IOL implantation group [11,36,37].

In the secondary IOL group, postoperative glaucoma developed in six (5.4%) eyes after the first surgery, and two (1.8%) after secondary IOL implantation. The incidence of glaucoma in patients with aphakia ranged from 0.9% to 32%, and lower rates were reported in pseudophakic patients [38,39]. Some authors reported that primary IOL implantation reduced the risk of early and intermediate-onset glaucoma. A theory suggested that IOL implantation could be a barrier for chemical material passage from the vitreous body to the anterior chamber and also supported the drainage angle in case of trabecular meshwork collapse [40,41]. Solmaz et al. reported the incidence of glaucoma as 33.3% in aphakic patients and 34.8% in patients with secondary IOL implantation, whereas they did not detect glaucoma in patients with primary IOL implantation [42]. Vasavada et al. reported glaucoma in 16% of aphakic patients and 14% of pseudophakic patients, and Solebo et al. reported 10 glaucoma in 23% of patients with aphakia and 10% of patients with pseudophakia [43,44]. Contrary to this view, before the IATS, IOLunder2 study group and Vasavada et al. did not find IOL implantation protective against glaucoma in their studies [43–45].

VAO is a major issue in congenital and developmental cataracts. In our study, VAO occurred in 3.6% of the secondary IOL group and 9.1% of the primary IOL group. Up to 100% opacifications have been reported if the posterior capsule is left intact and are more commonly reported in pseudophakic eyes than the aphakic eyes [36,43]. Van Looveren et al. reported clear visual axis after the bag-in-the-lens technique, and another in vitro study showed decreased epithelial proliferation with this technique [46]. Gimbel et al. and Stegmann developed different optic capture techniques for reducing VAO [47,48]. In the IATS, 68% of the patients in the primary IOL group and 14% of the patients in the contact lens group required surgery to clear the visual axis [7]. In a metaanalysis study, VAO was reported at a rate of 44% despite preventive techniques. As a result, many factors affect the incidence of VAO such as children’s age during the surgery, surgical technique, type of IOL, and the duration of steroid use [49].

In our study, IOL decentration and dislocation occurred in four (3.6%) eyes of the secondary IOL group and two (1.4%) eyes in the primary IOL group. All decentered IOLs were foldable 3-piece IOLs that had been implanted in the ciliary sulcus. We thought that decentration developed due to capsular phimosis or the size difference between the IOL and the fixation site. Trivedi et al. reported that axial length >23 mm was one of the important factors for IOL decentration and dislocation. They argued that long eyes might have wider anterior segments and sulcus distances, which might cause decentration [50]. They reported IOL decentration in four eyes (5.2%) and all decentration IOLs were foldable IOLs that had been implanted in the ciliary sulcus. None of their sulcus-placed PMMA IOLs developed decentration or dislocation. Therefore, they suggested optic capture for sulcus-placed foldable IOLs in the anterior and posterior capsulotomy to avoid decentration. Similarly, Wilson et al. placed nonfoldable IOLs in the sulcus to prevent decentration [51].

The retrospective design was one of the limitations in our study and the second was visual acuity outcomes depended on children’s age and the tests used.

In conclusion, the development of cataracts after the critical period for visual development seems to be the most important factor in childhood cataracts. Except for congenital presentation, unilaterality and the density of the cataract are also strong predictors of decreased visual acuity in this period. In this study, distance and near BCVA values were significantly better in the primary IOL group, which underwent surgery after 2 years of age. The rates of nystagmus and strabismus were significantly lower and the presence of binocular vision was more frequent than in the secondary IOL group. Long-term refraction outcomes and incidence of complications were similar in both primary and secondary IOL groups. Secondary IOL surgery time can be delayed to predict the refractive result and less high myopia probability in patients whose refraction is corrected using appropriate methods and who are compatible with occlusion therapy.

## Figures and Tables

**Table 1:** Demographic characteristics of the patients (*n* = 144).

	Groups		Test statistics	
	Congenital/developmental *n* = 64	Developmental *n* = 80	Test value	*p*-value

**Eye**				
Unilateral	17 (26.6)	29 (36.3)	χ^2^ = 1.535	0.281
Bilateral	47 (73.4)	51 (63.7)		

**Sex**, *n* (%)				
Boy	33 (51.6)	55 (68.8)	χ^2^ = 4.420	**0.040**
Girl	31 (48.4)	25 (31.2)		

**Age at last visit**, (*years*)				
*mean* ± *SD*	9.5 ± 4.5	12.8 ± 4.1	*T* = 4.606	**<0.001**
*min-max*	4.0–23.0	6.0–24.0		

**Age at surgery**, (years)				
*mean* ± *SD*	0.4 ± 0.3	5.9 ± 3.3	*T* = 14.602	**<0.001**
*min-max*	0.08–1.50	2.0–15.0		

**Follow-ups (years)**				
*M* (*IQR*)	8.0 (7.7)	5.0 (5.0)	*z =* 3.294	**0.001**
*min-max*	4.0–19.0	4.0–16.0		

**Preoperative strabismus** *n* (%)				
Yes	29 (45.3)	4 (5.0)	χ^2^ = 32.710	**<0.001**
No	35 (54.7)	76 (95.0)		

**Postoperative strabismus** *n* (%)				
Yes	33 (51.6)	20 (25.0)	χ^2^ = 10.786	**0.002**
No	31 (48.4)	60 (75.0)		

**Occlusion**, *n* (%)				
Yes	38 (59.4)	60 (75.0)	χ^2^ = 3.993	**0.049**
No	26 (40.6)	20 (25.0)		

**Nystagmus s**, *n* (%)				
No	35 (54.7)	77 (96.3)	χ^2^ = 35.536	**<0.001**
Yes	29 (45.3)	3 (3.7)		

**Stereopsis**, *n* (%)				
No	61 (95.3)	61 (76.3)	χ^2^ = 9.982	**0.002**
Yes	3 (4.7)	19 (23.7)		

**Secondary IOL surgery time** (years)	2.8 ± 0.4			
*mean* ± *SD*		-	-	-

*SD*: standard deviation, *M*: median, *IQR*: interquartile range, χ^2^: chi-square test, *t*: independent samples *t*-test, *z*: Mann-Whitney *U* test.

**Table 2:** Associations of postoperative nystagmus and strabismus with visual acuity (VA).

		Cataract			
		Developmental		Congenital/developmental	
**Postoperative VA**		**≤20/40**	**>20/40**	**≤20/40**	**>20/40**
**Nystagmus - postop**	(−)	12	65	8	27
	(+)	3	0	16	13
**Test statistics**	**Different**	**C2 = 13.506; p = 0.006** *		**C2 = 7.066; p = 0.010** *	
	**Correlation**	**r =−0.411; p = 0.0001** *		**r = ** **−** **0.331; p = 0.007** *	
**Strabismus - postop**	(−)	14	62	16	19
	(+)	1	3	8	21
**Test statistics**	**Different**	C2 = 0.108; p = 0.572 a		C2 = 2.224; p = 0.195 b	
	**Correlation**	r = −0.04; p = 0.746		r = 0.186; p = 0.140	

a Fisher’s exact test;

b Pearson’ s chi-square test; r: Pearson’ s correlation;

* statistically significant.

**Table 3:** Associations of nystagmus with strabismus.

	Developmental				Congenital/developmental			
Nystagmus	Strabismus (−)	Strabismus (+)	Test statistics		Strabismus (−)	Strabismus (+)	Test statistics	
	(n = 60)	(n = 20)	Test value	*p*-value	(n = 31)	(n = 33)	Test value	*p*-value
**Preop nystagmus (**−**)**	57	20	1.039	0.569^a^	22	13	6.430	0.011^b^
**Preop nystagmus (**−**)**	3	0			9	20		
**Postop nystagmus (**−**)**	58	20	0.684	0.999^a^	26	13	13.284	0.0001^b^
**Postop nystagmus (+)**	2	0			5	20		

a Fisher’s exact test;

b Pearson’ s chi-square test.

**Table 4:** Comparison of complications and additional intraocular surgeries.

	Groups	
	Congenital/developmental *n* = 111 (%)	Developmental *n* = 131 (%)
**Complications number**	30 (27.0)	27 (20.6)

**Number of eyes with complications**	31 (27.9)	24 (18.3)

Complications		
Membrane	10 (9.0)	7 (5.3)
Decentered IOL	4 (3.6)	1 (0.7)
IOL drop	0 (0.0)	1 (0.7)
PCO	4 (3.6)	12 (9.1)
Synechiae	4 (3.6)	3 (2.3)
Glaucoma	8 (7.2)	3 (2.3)

**Number of additional surgeries**	38 (34.2)	25 (19.1)

**Number of eyes needed additional surgery**	28 (25.2)	17 (12.9)

**Additional surgeries**		
Membranectomy	8 (7.2)	6 (4.5)
Synechiotomy capsulotomy	4 (3.6)	4 (3.1)
IOL correction	4 (3.6)	1 (0.7)
Vitrectomy + scleral IOL	1 (0.9)	1 (0.7)
Strabismus op	14 (12.6)	5 (3.8)
YAG capsulotomy	5 (4.5)	8 (6.1)
Trabeculectomy	2 (1.8)	0 (0.0)

**Table 5:** Comparison of preoperative and postoperative best-corrected visual acuity* (BCVA) and spherical equivalent (SE) (*n* = 242).

	Groups		Test statistics†	
	Congenital/developmental *n* = 111 *Mean* ± *SEM*	Developmental *n* = 131 *Mean*±*SEM*	*F* value	*p*-value
**SE**	−0.834 ± 0.410	−0.148 ± 0.415	1.379	0.242
**Preop distance BCVA**	-	1.363 ± 0.095	-	-
**Postop distance BCVA**	0.612±0.051	0.268 ± 0.041	27.624	**<0.001**
**Postop near BCVA**	0.450 ± 0.031	0.305 ± 2.1	14.69	**<0.001**
**Test statistics** ‡	-	*F* = 130.927; *p* < 0.001		

* Linear mixed model analysis; *SEM*: standard error of mean;

† postop comparison of groups;

‡ Comparison of preop-postop BCVA in the developmental group.

**Table 6:** Comparison of myopic shift in groups^*^ (*n* = 242).

	Groups		Test statistics	

	Congenital/developmental *n* (%)	Developmental *n* (%)	χ^2^ value	*p*-value

**Hyperopia**	47 (42.3)	67 (51.1)	1.869	0.172
**Myopia**	64 (57.7)	64 (48.9)		

χ^2^: chi-square test statistic.

**Table 7:** Comparison of SE in operated unilateral eyes with other healthy eyes.

	Eye		Test statistics	
	Operated *Mean* ± SEM	Healthy *Mean* ± SM	Test value	*p*-value
**Congenital/developmental**	−1.074 ± 0.868	0.927 ± 0.469	*t* = 2.788	0.013
**Developmental**	−0.353 ± 0.521	0.629 ± 1.049	*t* = 2.059	0.049

*SEM*: standard error of mean; *t*: paired *t*-test.
